# Susceptibility reporting and antibiotic prescribing for UTIs in the inpatient setting: a nudge toward improved stewardship

**DOI:** 10.1017/ash.2025.10159

**Published:** 2025-10-08

**Authors:** Madison G. Ponder, Kevin Alby, Lindsay M. Daniels, Ashlyn M. Norris, Alan C. Kinlaw

**Affiliations:** 1 Gillings School of Global Public Health, Department of Epidemiology, University of North Carolina at Chapel Hill, Chapel Hill, NC, USA; 2 Department of Pharmacy, University of North Carolina Medical Center, Chapel Hill, NC, USA; 3 Department of Pathology and Laboratory Medicine, University of North Carolina Medical Center, Chapel Hill, NC, USA; 4 School of Pharmacy, Division of Pharmaceutical Outcomes and Policy, University of North Carolina at Chapel Hill, Chapel Hill, NC, USA

## Introduction

Urinary tract infections (UTIs) are common infections, particularly among women and older adults.^1^ Antimicrobial resistance among UTI pathogens is increasing, making them key targets for antimicrobial stewardship programs.^2,3^ Electronic health records (EHRs) can provide decision support to guide stewardship efforts and antibiotic prescribing.^4^

We evaluated the impact of a low-resource EHR-based intervention where cephalexin was added to the susceptibility profile for urine culture results from inpatient, observation, and emergency settings based on inferred susceptibility of cefazolin for uncomplicated UTIs.^5–7^

## Methods

### Study population

We included patients age ≥18 years admitted to inpatient, observation, or emergency settings at University of North Carolina Medical Center with urine cultures positive for *Escherichia coli*, *Klebsiella pneumonia*, and/or *Proteus mirabilis* who received ≥1 antibiotic indicated for UTI. All settings could order a urinalysis or urine culture separately, while only the emergency department could also order a urinalysis to reflex urine culture. The pre and postintervention periods were September 1, 2018–September 9, 2019 and September 10, 2019–March 11, 2020, respectively. We excluded patients with: (1) specimen collection >15 days after admission and (2) susceptibility results returned ≥5 days after specimen collection (Supplemental Figure 1).

To assess the effect of the intervention on prescribing in response to timely availability of culture results, we examined medication orders occurring until discharge (not exceeding 7 d after culture susceptibility results). Patients were classified as “result not used” if ≥5 days passed between susceptibility result availability and any antibiotic prescription, or if discharged before results became available.

### Intervention

On September 10, 2019, cephalexin was added to EHR susceptibility profiles for all urine cultures positive for *Escherichia coli*, *Klebsiella pneumonia*, and/or *Proteus mirabilis* with susceptibility testing for cefazolin. A comment on the susceptibility profile noted that cephalexin is recommended for uncomplicated cases only (Supplemental Figure 2). No announcement or training accompanied the intervention, but prospective audit-and-feedback was conducted by the antimicrobial stewardship team routinely. Monthly prescription prevalence was calculated as the number of patients prescribed each antibiotic after susceptibility results became available out of all eligible patients that month.

### Interrupted time series statistical analysis

To evaluate the impact of the intervention on prescribing (shown by trend deflections), we used segmented linear regression to model interrupted time series data for monthly prescription prevalence. Our models accounted for seasonality using a sinusoidal function^8^ and autocorrelated errors using lagged model parameters based on Durbin-Watson alpha of 0.3.^9^

This study was approved by the University of North Carolina institutional review board. Analyses were performed using SAS 9.4 (SAS Institute, Cary, NC); Figures were created using R version 4.4.3 (R Core Team, Vienna, Austria) and RStudio version 2024.12.1.563 (Posit Team, Boston, MA).

## Results

In total, 1981 UTIs were treated among 1788 unique patients (preintervention: *n* = 1 342; postintervention: *n* = 639). The median length of stay for both intervention periods was 2 days (interquartile range (IQR): 0–5 d). Among patients who were inpatient or observation stays, the median was 4 days (IQR: 2–7 d). Half (49.1%) of patients were discharged before culture results were returned; 70.0% of these were emergency department patients. Most patients were treated empirically with ceftriaxone (preintervention: 49.0%; postintervention: 53.4%).

**Figure 1.**
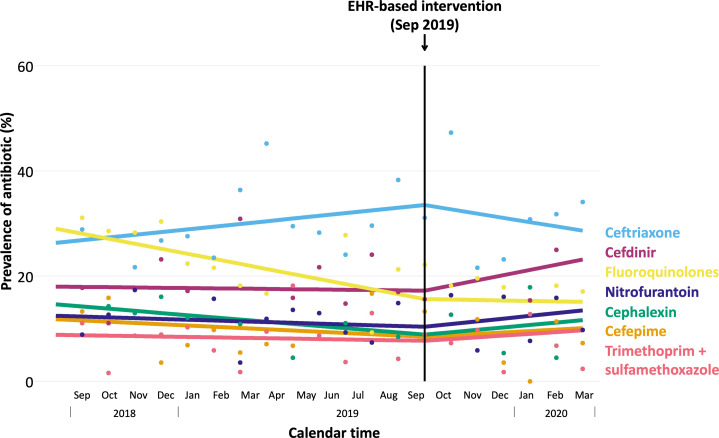
Interrupted time series analysis of antibiotic prescribing by medication for urinary tract infection after results from urine culture with susceptibilities. The interrupted time series includes one inflection point at the intervention timepoint of September 2019. Patients could have multiple prescriptions of antibiotics, meaning prevalence measures for each antibiotic combined could be greater than 100%.

At the beginning of the study period, fluoroquinolones had the highest prescription prevalence (29.0%) compared to 14.7% for cephalexin, which was the targeted antibiotic for this intervention (Figure 1; Supplemental Table 1).

Before the intervention, cephalexin prevalence decreased by 0.4% (95%CI: –0.6%, –0.3%) per month. Afterward, this trend deflected upward (*P* = .01), increasing by 0.5% (95%CI: 0.1%, 0.9%) per month. Preintervention fluoroquinolone prevalence decreased by 1.0% per month (95%CI: –1.2%, –0.9%) but stabilized postintervention (*P* = .01). Ceftriaxone prevalence increased 0.6% (95%CI: 0.0%, 1.1%) per month before the intervention but negligibly decreased by –0.9% (95%CI: –2.5%, 0.8%) afterward. Nitrofurantoin increased modestly in prevalence by 0.6% (95%CI: 0.2%, 1.0%) per month postintervention (*P* = .03). Cefdinir negligibly increased by 1.1% (95%CI: –0.1%, 2.2%) per month postintervention (*P* = .18).

## Discussion

This analysis demonstrates a modest increase in cephalexin use following a subtle, low-resource EHR-based intervention on antibiotic prescribing patterns for UTIs. While small, this change highlights potential for a greater impact with additional provider engagement or resource-intensive strategies.

Almost half of patients either received no medication within 5 days following culture result availability or were discharged before. This was most common among emergency department patients, whose stays were often shorter than the typical turnaround time. This may have led to susceptibility results going unused in decision-making for many patients and may relate to urinalysis to reflex cultures being available for order in the emergency department.

The prevalence of fluoroquinolones declined prior to the intervention, potentially related to an increased awareness of resistance and adverse reactions to fluoroquinolones in the medical community. Aside from prospective audit-and-feedback, no other interventions were in effect during the study periods.

UTI indications did not distinguish between complicated or uncomplicated cases. Cephalexin is recommended for uncomplicated UTIs, with unclear efficacy in complicated cases.^10^ Cephalexin may have been avoided for uncomplicated cases, likely leading to a weaker observed impact. We also could not determine if UTIs was recurrent; about 10% of patients had multiple UTIs during the study period. Notably, resistance to the oral cephalosporins, based on the cefazolin urine surrogate breakpoint, remained stable and consistently low (≤10%) for 2019 and 2020.

EHR-based interventions like this one can have meaningful impact on practice and given their low-resource use, they may warrant broader implementation. Similar efforts in outpatient settings or with other antibiotics, such as inferring doxycycline susceptibility from tetracycline, could expand stewardship impact.^7^

## Supporting information

10.1017/ash.2025.10159.sm001 Ponder et al. supplementary material Ponder et al. supplementary material

## Research transparency and reproducibility

Data sources are not publicly available. Analytic code is available upon request.
