# Supplementary material for: Susceptibility reporting and antibiotic prescribing for UTIs in the inpatient setting: a nudge toward improved stewardship
Source: Antimicrob Steward Healthc Epidemiol. 2025 Oct 8;5(1):e254. doi: 10.1017/ash.2025.10159 (PMC12538342; doi:10.1017/ash.2025.10159)
Supplement: Ponder et al. supplementary material [file S2732494X25101599sup001.docx]

**Supplemental Figure 1**: Patients were assessed for inclusion into the study. Of the 2691 patients eligible for assessment, 1981 were included into our pre- or post-intervention exposed group.
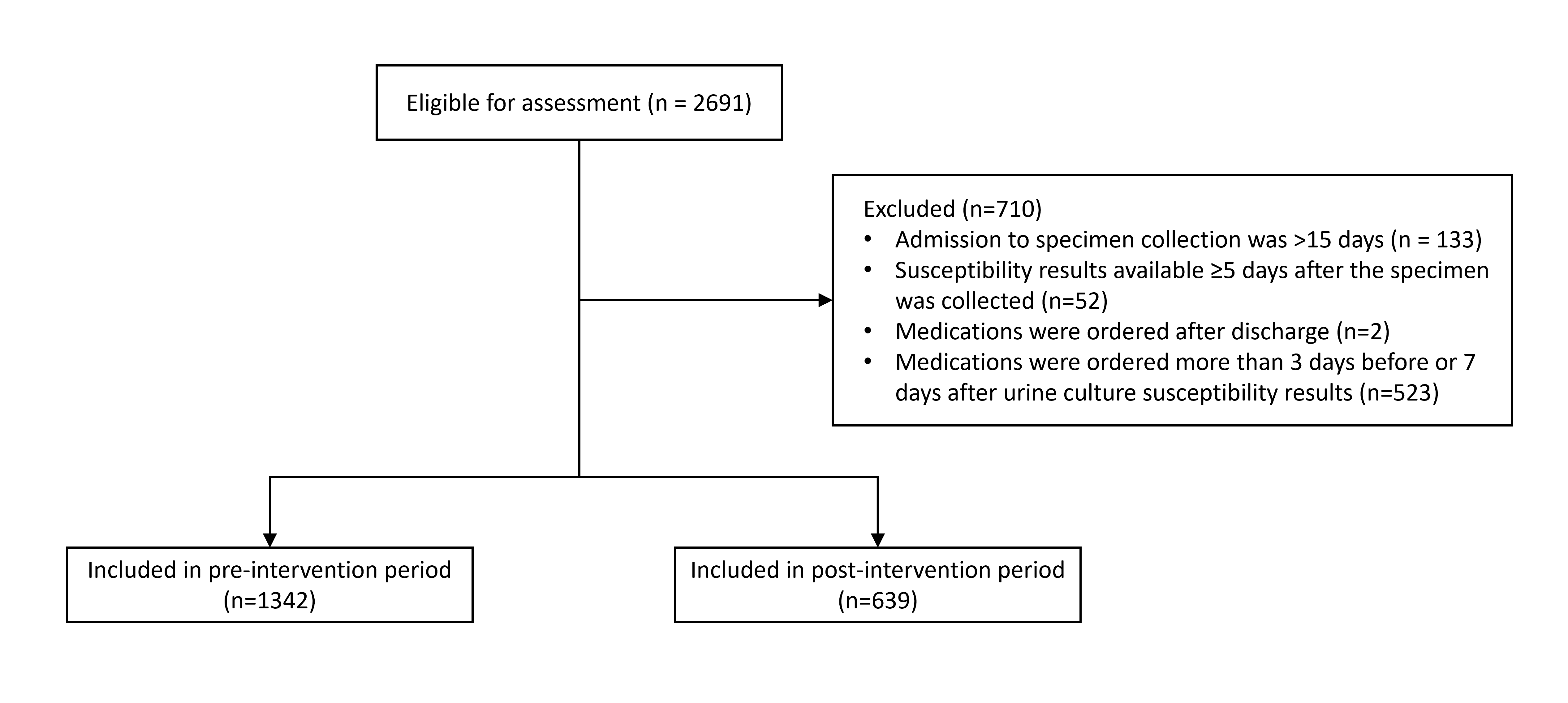


**Supplemental Figure 2**: Example antibiotic susceptibility profiles before and after EHR-based intervention.

**
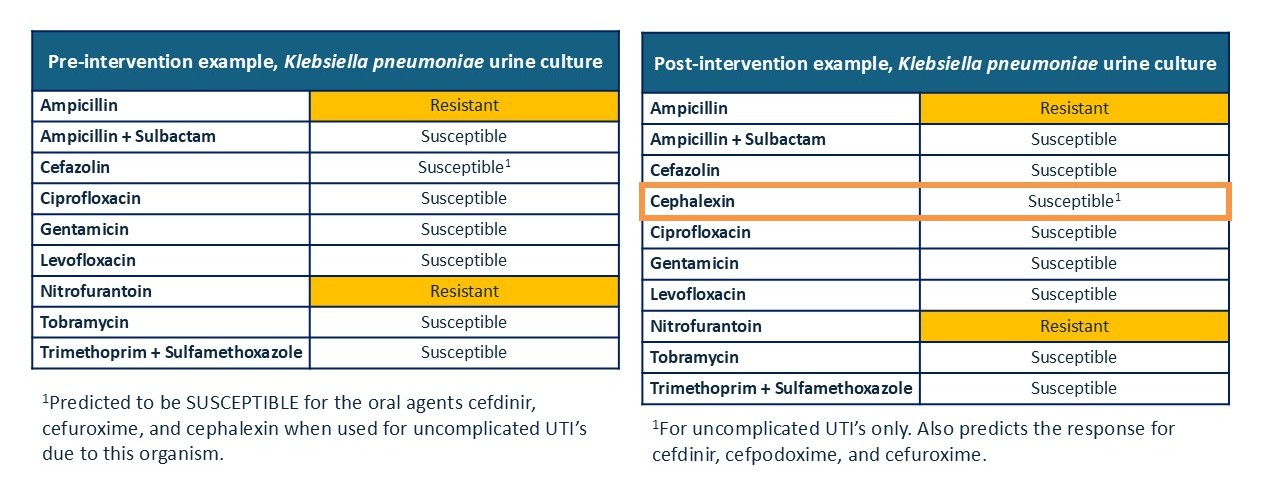
**

**Supplemental Table 1**: Interrupted time series results of prescription prevalence for antibiotics* used to treat urinary tract infections. The baseline prescription prevalence is the estimated prevalence of each antibiotic at the start of the study period (September 2018). The baseline trend estimates the change in prescription prevalences per month up until the intervention. The hinge estimates the deflection per month in the baseline trend, or the instantaneous change from the pre-intervention trend to the post-intervention trend. The post-intervention trend estimates the change in prescription prevalences per month immediately following the intervention through the end of the study period (March 2020).

|  |  |  | Baseline Trend | |  | Hinge | | |  | Post-Intervention Trend | |
| --- | --- | --- | --- | --- | --- | --- | --- | --- | --- | --- | --- |
| Antibiotic | Baseline Prescription Prevalence |  | Trend Estimate | 95% CI |  | Deflection in Trend | 95% CI | p-value |  | Trend Estimate | 95% CI |
|  |  |  |  |  |  |  |  |  |  |  |  |
| Cephalexin | 14.7 |  | -0.4 | (-0.6, -0.3) |  | 0.9 | (0.4, 1.5) | 0.01 |  | 0.5 | (0.1, 0.9) |
| Fluoroquinolone | 29.0 |  | -1.0 | (-1.2, -0.9) |  | 0.9 | (0.4, 1.5) | 0.01 |  | -0.1 | (-0.5, 0.4) |
| Cefdinir | 18.0 |  | -0.1 | (-0.4, 0.3) |  | 1.1 | (-0.4, 2.6) | 0.18 |  | 1.1 | (-0.1, 2.2) |
| Ceftriaxone | 26.3 |  | 0.6 | (0.0, 1.1) |  | -1.4 | (-3.5, 0.6) | 0.19 |  | -0.9 | (-2.5, 0.8) |
| Cefepime | 11.8 |  | -0.3 | (-0.6, 0.0) |  | 0.6 | (-0.8, 1.9) | 0.43 |  | 0.3 | (-0.7, 1.3) |
| Nitrofurantoin | 12.5 |  | -0.2 | (-0.3, 0.0) |  | 0.7 | (0.2, 1.3) | 0.03 |  | 0.6 | (0.2, 1.0) |
| Trimethoprim/  sulfamethoxazole | 8.9 |  | -0.1 | (-0.3, 0.1) |  | 0.4 | (-0.4, 1.3) | 0.36 |  | 0.4 | (-0.4, 1.1) |
|  |  |  |  |  |  |  |  |  |  |  |  |

Note. CI: confidence interval.

*Other antibiotics may have been prescribed during this study period but were not included in the analysis due to sparse data
